# Preserving Gadolinium Speciation in Environmental Waters: Establishing Hold Times and Storage Protocols for Reliable Analysis

**DOI:** 10.1021/acsestwater.6c00241

**Published:** 2026-05-26

**Authors:** Ahmad Ezzaldine, Malsha Kanaththage, Gayan Rubasinghege, Bonnie Frey, G. Patricia Escobar, Rachel Coyte

**Affiliations:** † Department of Earth and Environmental Science, 7374 New Mexico Institute of Mining and Technology, Socorro, New Mexico 87801, United States; ‡ Department of Chemistry, New Mexico Institute of Mining and Technology, Socorro, New Mexico 87801, United States; § New Mexico Bureau of Geology and Mineral Resources, Socorro, New Mexico 87801, United States; ∥ Kidney Institute of New Mexico, 12289 University of New Mexico Health Sciences Center, Albuquerque, New Mexico 87106, United States; ⊥ Research Service, New Mexico Veterans Administration Health Care System, Albuquerque, New Mexico 87106, United States

**Keywords:** gadolinium contamination, gadolinium-based contrast agents (GBCAs), gadolinium speciation, sample preservation conditions, speciation preservation protocol, environmental water samples

## Abstract

Gadolinium (Gd) has emerged as a trace contaminant in aquatic environments due to the widespread use of gadolinium-based contrast agents (GBCAs) in magnetic resonance imaging (MRI) diagnostics. This study evaluates how preservation conditions, including temperature, acidification, and filtration, affect the stability of Gd chelates in three water matrices (deionized water, tap water, and river water) using inductively coupled plasma mass spectrometry (ICP-MS) for total Gd quantification and ion chromatography coupled with ICP-MS (IC-ICP-MS) for individual GBCA quantification. Across all experiments, the linear GBCA was the most susceptible to degradation, with acidification and ion-rich waters accelerating their dissociation, while macrocyclic agents remained more stable. Freezing provided no preservation benefit and sometimes introduced artifacts possibly related to freeze-concentration effects. Filtration improved recoveries in river water by reducing interactions with particulates and microbial activity, and refrigeration slowed degradation but did not fully prevent it in complex matrices. These patterns show that certain preservation choices can alter apparent speciation and lead to underestimation of linear chelates and misinterpretation of GBCA sources. The results provide standardized preservation protocols for sample handling, including avoiding acidification, minimizing storage time, and refrigerating samples when immediate analysis is not possible.

## Graphical abstract

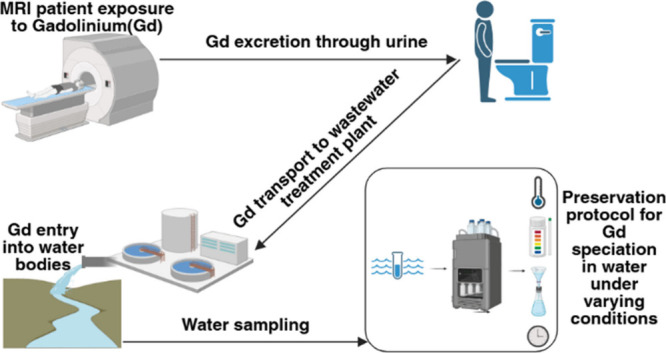

## Introduction

Gadolinium (Gd), a rare earth element (REE), has a wide range of applications, including electronics, alloys, specialty glass, and energy, as well as medical imaging.^1^ Its most widespread use is in medical diagnostics, where it has been used since the 1980s to improve magnetic resonance imaging (MRI).^2–4^ The strong paramagnetic properties of Gd enhance MRI signals by shortening the relaxation time constants of adjacent hydrogen nuclei.^5^ In clinical formulations, Gd^3+^ is chelated with polyaminocarboxylic ligands to prevent toxic effects, forming thermodynamically stable complexes known as GBCAs.^6–8^

After administration to patients, GBCAs are excreted mostly intact in the patient’s urine and subsequently enter wastewater streams. GBCAs are generally not effectively removed by conventional wastewater treatment plants (WWTPs) and are discharged into the environment, where they can enter drinking water supplies.^9,10^ Anthropogenic Gd is reported to be a key emerging contaminant in regions with high MRI usage,^11,12^ and anthropogenic Gd pollution related to MRIs have been reported in water bodies in Europe, North America, South America, Australia and Asia.^13–22^ Of primary environmental concern is the potential for GBCAs to dissociate under certain conditions and release Gd^3+^, which poses documented health risks, including renal damage, cognitive impairments, and gastrointestinal disorders, particularly in populations already vulnerable due to preexisting health conditions.^23,24^

Anthropogenic Gd concentrations in natural waters are often inferred from neighboring REE concentrations, assuming that Gd follows a continuous REE pattern and that any positive anomaly is attributed to human input. Linear, logarithmic, geometric, and polynomial models have been used, each differing in reference elements and assumptions about Gd behavior.^6,13,25–28^ Statistical approaches have also been developed to estimate geogenic Gd background without relying on neighboring REEs. These include iterative population reduction and probability plots, which separate natural background from anthropogenic input using measured concentration distributions.^29^ These methods quantify total anthropogenic Gd but cannot distinguish intact GBCAs from other forms of Gd, a fundamental gap that obscures the mechanistic understanding needed to predict environmental fate, evaluates transformation pathways, and assess actual toxicological risk.

Methods for quantifying GBCAs emerged in the early 2000s, using ion-exclusion and size-exclusion chromatography coupled to ICP-MS, initially applied to deionized water and laboratory-prepared solutions.^30,31^ These techniques were soon extended to clinical matrices, including urine, plasma, hair, and other patient-derived fluids.^32,33^ Subsequent environmental studies employed capillary electrophoresis and ultrafiltration to examine Gd interactions with natural organic matter.^34,35^ The coupling of hydrophilic interaction liquid chromatography (HILIC) with MS enabled simultaneous separation of multiple commercial agents in complex samples such as hospital effluents and sewage.^17,36^ Over the following decade, applications broadened to include wastewater, sewage sludge, rivers, aquatic plants, and drinking water.^10,37,38^ Sensitivity improved markedly, with detection limits reaching the pmol L^–1^ range through innovations such as high-resolution sector field ICP-MS, desolvation systems, and ultrasonic nebulization.^39,40^ More recent developments emphasize analytical efficiency and automation, including rapid ion chromatography and fully automated IC–ICP-MS platforms capable of quantifying emerging agents such as Vueway at picomolar levels.^41,42^

Across the literature, there is substantial variability in sample handling for Gd speciation analysis, with storage conditions, preservation methods, and matrix-specific considerations often reported inconsistently or incompletely within method sections. A summary of preservation methods reported in previous Gd speciation studies across different environments is provided in Table S1 (Supporting Information). The absence of systematic evaluation of these parameters introduces potential artifacts and limits the comparability of reported speciation data across studies. This research addresses that gap by systematically assessing preservation conditions (temperature, pH, and filtration) and their influence on GBCA stability in water samples. Omniscan, Gadovist, Dotarem, and Vueway were chosen as representative GBCAs to cover both linear and macrocyclic agents, as well as established and emerging clinical formulations. Studies have found that the dominant anthropogenic species of Gd in natural waters are typically Gadovist, Dotarem, and Magnevist, whereas linear agents such as Omniscan and Multihance are rarely detected or occur only at trace levels.^36,37,39,41^ The findings also define the time scales over which these factors become critical, establishing practical hold times for reliable measurements. The findings will provide the basis for a standardized protocol for preserving Gd speciation in natural waters, and offer a framework that future studies may adopt and refine to achieve greater consistency and comparability in speciation research.

## Methods

### Water Matrices and Sample Preparation

In this study, four GBCAs, Gadovist, Vueway, Dotarem, and Omniscan, were evaluated in three distinct water matrices: (i) deionized (DI) water, to represent a baseline organic-free and ion-free system; (ii) municipal tap water, to reflect treated urban supplies; and (iii) Rio Grande River water, a natural surface water matrix characterized by a more complex geochemistry including organic matter and suspended particulates (Figure 1).

**Figure 1:**
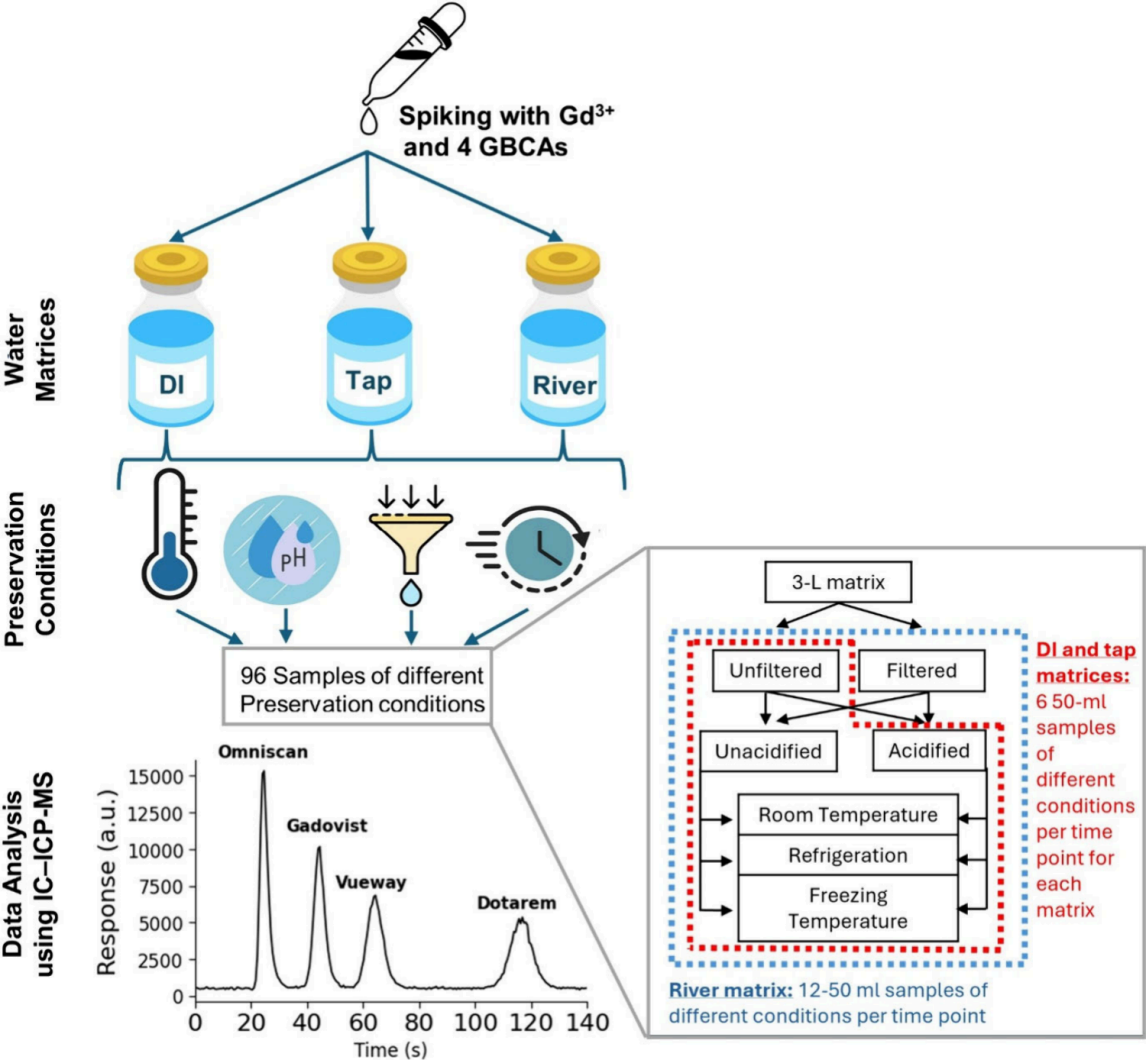
Experimental workflow showing preservation treatments applied to GBCAs in river, tap, and DI (deionized) water.

For each water matrix, 3-L bulk samples were prepared and spiked with free Gd^3+^ and the four clinically used GBCAs to achieve a final concentration of 5 μg/L for each compound.

### Preservation Treatments

Two preservation-related factors were tested: temperature, and acidification for all water matrices. Temperature conditions included refrigeration at 4 °C to represent the typical cold storage used in laboratories, room temperature, and freezing at −20 °C, followed by thawing at room temperature, a procedure standard in GBCA speciation analysis research. From the bulk sample, 96 subsamples were prepared, half of which were acidified to pH < 2 by adding concentrated ultrapure nitric acid. Acidification to a pH < 2 is a standard practice for the analysis of dissolved trace elements in water, including total Gd.^43^

A third factor, filtration, was studied only on river water samples, where suspended solids, colloids, and microbiota are more abundant and may influence Gd speciation. Samples were filtered through 0.45 μm poly­(ether sulfone) (PES) membranes into the PP centrifuge tubes. Filtration, which is standard in many trace metal protocols, removes particulates and colloidal material that may bind metals or metal–ligand complexes, thus, isolating the dissolved metals fraction from metals held on these suspended solids. Filtration also prevents microbial activity from altering the water chemistry.^44^

Each preservation condition was applied across the three different water types and sample splits were taken at four time points for analysis: 24 h, 1 week, 4 weeks, and 2 months (Figure 1). This produced 96 samples in total: 48 for the river matrix (12 preservation conditions × 4 time points), and 24 each for the municipal tap and DI matrices (6 conditions × 4 time points). Detailed descriptions of the preservation conditions are provided in Figure S1 (Supporting Information).

### IC-ICP-MS and ICP-MS Instrumentation Overview

IC–ICP-MS instrumentation and Gd speciation analysis were performed using a prepFAST IC system from Elemental Scientific Inc. (Omaha, NE, USA) for sample dilution and chromatographic separation. The system was equipped with a 1.5 mL sample dilution loop and a polymer-based anion-exchange column (CF-Gd-01, 50 × 4 mm) functionalized with quaternary ammonium alkyl groups for GBCA speciation analysis. A 200 μL injection loop was used to introduce samples diluted in ultrapure Milli-Q water. The prepFAST IC system was directly coupled to an Agilent 7900 ICP-MS for the detection of separated GBCAs. Detection was performed by ICP-MS, monitoring the mass-to-charge ratios of ^157^Gd and ^158^Gd, with ^157^Gd used for quantification. Total Gd was measured using the ICP-MS instrument, which is capable of analyzing 13 REEs, with Rh, In, and Ir as internal standards and a target recovery of 85–105%.

GBCA’s recovery percentage was then calculated by comparing the measured GBCA concentration of each sample to the expected value of about 5 μg/L.

Details of sampling information, GBCA selection and preparation, chromatographic conditions, calibration and data processing, and recovery experiment design, method limits of detection (LOD) and quantification (LOQ), and analytical precision for both IC–ICP-MS and ICP-MS analyses are provided in Sections S1–S6, Figure S2, and Tables S2–S**4** (Supporting Information).

### Statistical Analysis

Statistical analysis was performed on the data set to evaluate GBCA recovery. Each sample was analyzed in three analytical replicates, and mean recovery values and standard deviations were calculated for acidified versus unacidified samples. The Mann–Whitney U test,^15^ a nonparametric alternative to the independent *t* test, was used to examine whether acidification affected recovery values for each GBCA over time. This test was chosen because it does not assume normality and is appropriate for small sample sizes. For each GBCA, independent Mann–Whitney U tests were conducted comparing acidified and unacidified samples. To control the family wise error rate across multiple comparisons, a Bonferroni correction was applied (Dunn, 1961),^100^ yielding a corrected significance threshold of α = 0.05/n, where n is the number of comparisons. Tests with Bonferroni-adjusted *p* < 0.05 were considered statistically significant. Similarly, the effect of filtration on river water samples was assessed by pooling all time points and comparing filtered versus unfiltered river samples for each GBCA using Mann–Whitney U tests. The influence of temperature on GBCA recovery was evaluated by pooling all time points and comparing the three temperature conditions using Kruskal–Wallis tests.^18^ A nonparametric test different from Mann–Whitney U was used because there were more than two groups, and Kruskal–Wallis accommodates multigroup comparisons without assuming normality.

## Results and Discussion

To understand how different storage and handling conditions influence GBCAs, their stability across a range of water types and preservation methods was evaluated. The sections below summarize the main findings, highlight patterns observed for each factor, provide guidance on preservation protocols, and describe implications for environmental monitoring and future research.

### Effect of Acidification

Samples were classified to compare acidified and unacidified speciation measurements for each GBCA across time. Acidification to pH < 2 caused a marked decrease in recovery of GBCAs, and the extent of degradation depended strongly on chelate structure (Figure 2). Omniscan, the only linear chelate in the group, recovered to nearly 2% within 24 h under acidified conditions and remained almost null throughout the full 8-week period. Under unacidified conditions, Omniscan declined more slowly, from about 94% at 24 h to 33% after 8 weeks. Acidification, therefore, accelerated its degradation substantially. Gadovist and Dotarem, both macrocyclic agents, kept 82–110% recovery in unacidified samples throughout the experiment. Under acidified conditions, both decreased over time: Gadovist dropped from almost complete recovery at 24 h to about 26% after 8 weeks, and Dotarem followed a similar trend. Vueway showed the highest acid stability among the macrocyclic agents, with about 72% recovery after 8 weeks of acidified storage. Overall, acidification produced clear, time-dependent losses that followed the stability order Omniscan (linear) ≫ Gadovist ≈ Dotarem > Vueway.

**Figure 2:**
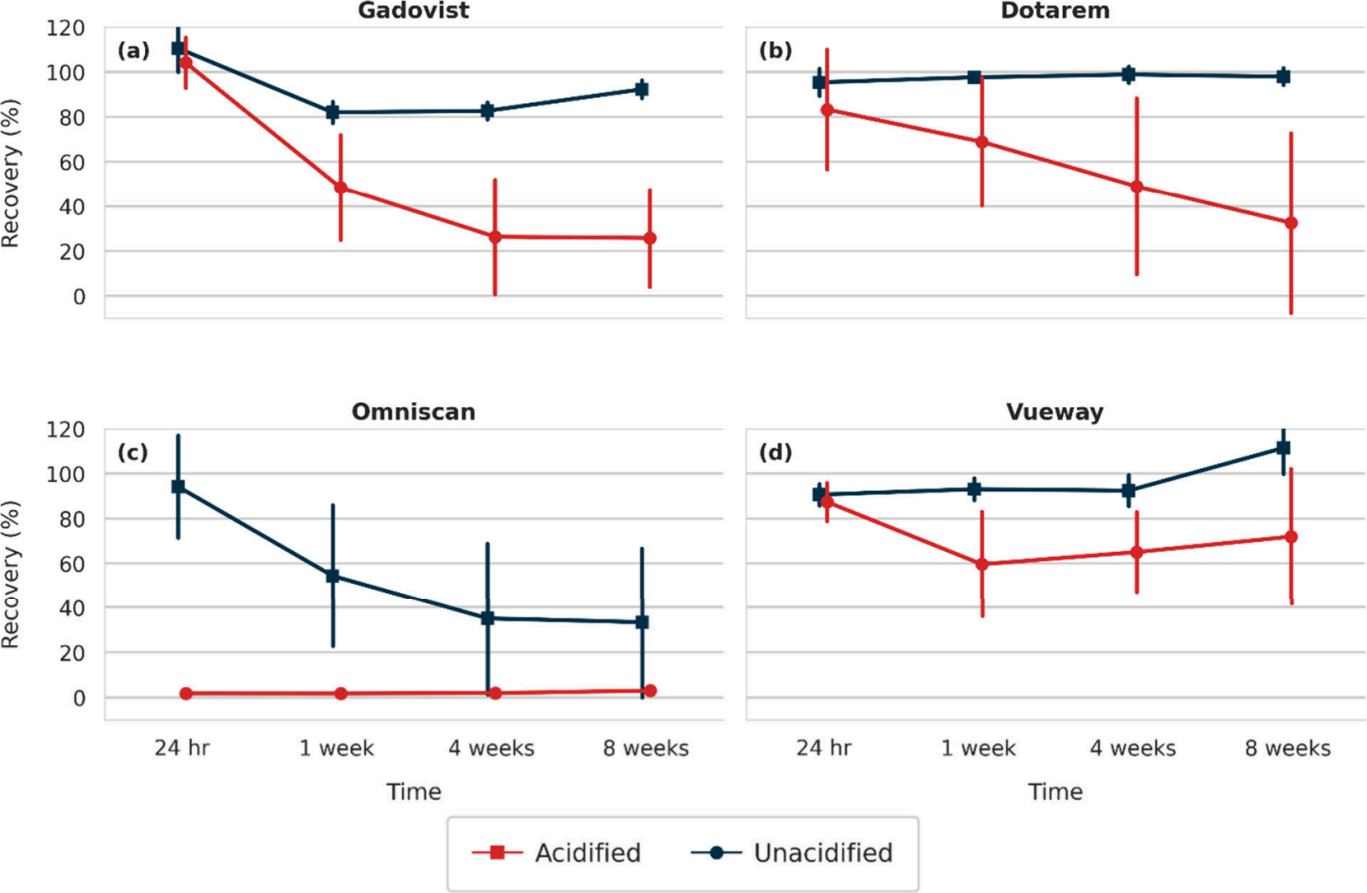
Recovery of GBCAs over time under acidified and unacidified storage conditions. Points represent mean recovery (%) from pooled measurements, and error bars show the standard deviation. Acidification caused time-dependent losses that differed by chelate structure, with the linear agent Omniscan degrading rapidly and macrocyclic agents (Gadovist, Dotarem, and Vueway) showing greater resistance to degradation.

These results demonstrate that acidification does not affect GBCAs equally. The response depends strongly on chelate structure. Omniscan, a linear chelate with low stability, is highly prone to dissociation at low pH. Macrocyclic chelates are more resistant and maintain their integrity under acidification, remaining stable for short durations but gradually degrading during long-term storage. This aligns with earlier speciation studies showing that Magnevist (linear chelate) complexes dissociate below pH 3 while remaining stable at neutral conditions, leading to selective retention of free Gd^3+^ but masking intact chelates.^30^ Similar concerns about method-induced artifacts have been noted in biological matrices, where extraction under alkaline or acidic conditions caused partial degradation of Magnevist into free Gd^3+^.^32^ Differences in thermodynamic stability (log K) among GBCAs, which are generally higher for macrocyclic agents such as Dotarem (25.6) and Gadovist (21.8), intermediate for Vueway (19.7), and lower for linear agents such as Magnevist (22.5) and Omniscan (16.9), can provide a convenient reference point, even though thermodynamic properties alone do not capture all aspects of complex behavior.^45,46^

In DI water, where matrix interference is minimal, the acidified sample after 1 week shows greater loss of GBCAs than the unacidified sample after 8 weeks, with total Gd remaining equal (Samples A and B, Figure 3). This indicates an immediate effect of acidification compared to unacidified storage.

**Figure 3:**
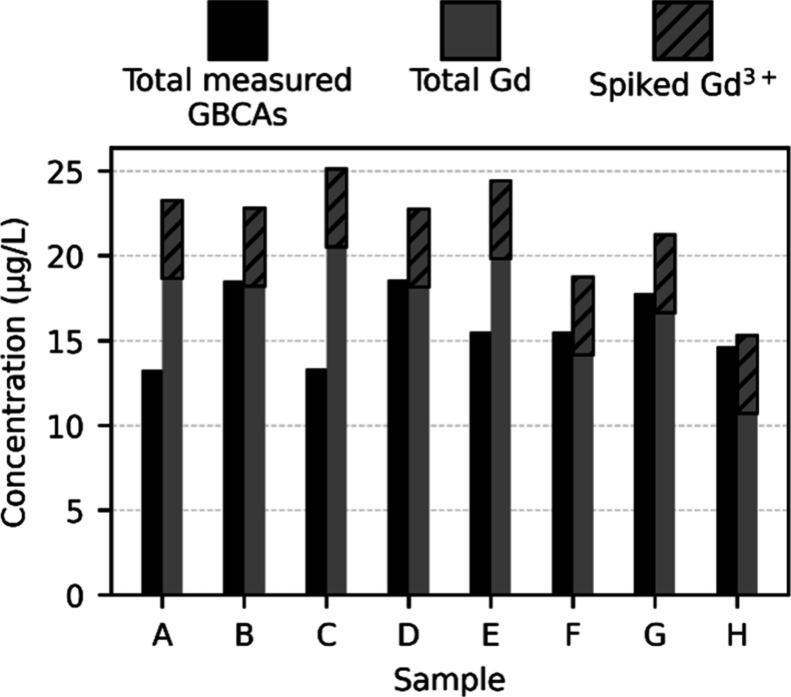
Measured GBCAs and total gadolinium in selected water samples. The expected balance corresponds to ∼20 μg/L for total GBCAs and ∼25 μg/L for total Gd; better preservation is reflected when recoveries approach these values. *X*-axis labels (from left to right): (A) deionized, room temperature, acidified, 1 week; (B) deionized, room temperature, unacidified, 8 weeks; (C) deionized, freezing, unacidified, 4 weeks; (d) deionized, freezing, unacidified, 8 weeks; (E) tap, room temperature, unacidified, 8 weeks; (F) river, room temperature, unacidified, filtered, 8 weeks; (G) river, 4°C, unacidified, filtered, 8 weeks; and (H) river, freezing, unacidified, filtered, 8 weeks.

To assess whether the observed differences between acidified and unacidified samples were statistically significant, Mann–Whitney U tests were performed for each GBCA at 24 h and 8 weeks. The U statistic shows how separate the two groups are based on ranked values. A smaller U indicates that the acidified samples had consistently lower recoveries than the unacidified ones. At 24 h, Omniscan showed complete separation between treatments (U = 0.0, Bonferroni-adjusted p = 0.00015). This supports the immediate loss under acid conditions. Gadovist (U = 45.0, p = 0.50), Vueway (U = 56.0, p = 1.00), and Dotarem (U = 39.0, p = 0.24) showed no significant difference at that time. After 8weeks, Gadovist (U = 0.0, p = 0.00015) and Dotarem (U = 0.0, p = 0.00015) showed strong evidence of acid-related loss. Vueway (U = 19.0, p = 0.0097) also showed a smaller but still noticeable effect. Pooling by other factors showed no significant relationships; the detailed test results are provided in the Supporting Information.

Acidification is often used to preserve water samples for trace-metal analysis, but in Gd speciation, it compromises sample integrity. Selective degradation of specific chelates alters the apparent distribution of anthropogenic Gd. The low recovery problem becomes more pronounced over time, since even macrocyclic GBCAs may undergo significant losses during extended storage, leading to further underestimation of their presence.

Because of this selective degradation, acidification is not an appropriate method for quantifying anthropogenic Gd inputs, especially when analysis is delayed. For this reason, acidified samples were excluded from further analysis under the following experimental conditions.

### Behavior of GBCAs in Simple Matrices (DI and Tap Water) at Three Storage Temperatures

Room temperature, refrigeration, and freezing produced similar results for DI and tap water. Macrocyclic agents were stable, maintaining recoveries between 80–100% (Figure 4, Figure 5).

**Figure 4:**
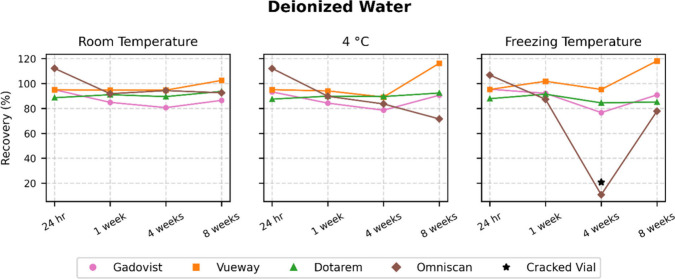
Stability of unacidified GBCAs in DI over time and storage temperatures. Macrocyclic agents (Gadovist, Vueway, and Dotarem) remained largely stable, while the linear agent Omniscan showed a slow, continuous decline. The asterisk at week 4 under freezing conditions denotes a cracked sample vial.

**Figure 5:**
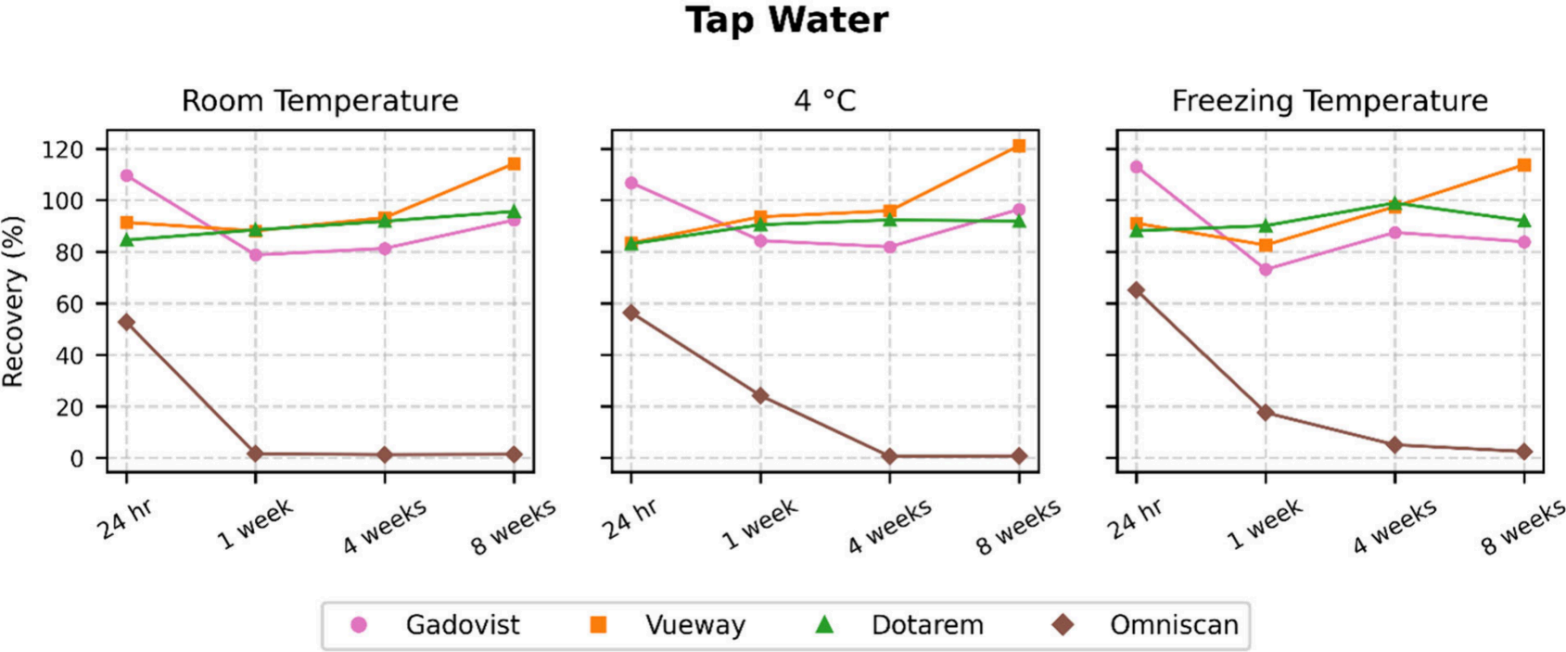
Stability of unacidified GBCAs agents in tap water over time and storage temperatures. Omniscan showed rapid loss across all temperatures, whereas macrocyclic agents remained largely stable throughout the 8 weeks.

### Deionized Water

In DI water, all four agents had high recoveries (90–110%) within the first week. Over the full 8-week period, the macrocyclic GBCAs (Gadovist, Vueway, and Dotarem) behaved similarly and remained largely stable with slow, modest declines whether frozen, refrigerated or at room temperature. By Week 8, recoveries generally trended toward 80–90%. In contrast, concentrations of Omniscan showed a slightly more continuous, though still slow, decline during storage (Figure 4). This likely reflects minor hydrolysis or adsorption in the absence of competing ions in the DI matrix.

### Tap Water

In tap water, differences between macrocyclic and linear agents were more pronounced. Omniscan degraded rapidly, with recoveries ranging from 0% to 20% within the first week, regardless of storage temperature (Figure 5). Notably, after 8 weeks of storage, total Gd remained in solution while total GBCAs were substantially reduced (Sample E in Figure 3), mainly due to Omniscan degradation. This decline could be related to a transmetalation process, where divalent cations such as Ca^2+^ and Mg^2+^ in tap water can displace Gd^3+^ from the linear ligand through competition. Once released, Gd^3+^ is no longer part of the Omniscan molecule, which results in the apparent loss.

In contrast, macrocyclic agents were more stable. Dotarem and Vueway maintained recovery rates of 85–95%, while Gadovist dipped slightly between the first and the fourth week before stabilizing. No significant differences were observed between room-temperature, refrigerated, or frozen samples. Birka et al. (2016) studied real drinking water systems and found that only macrocyclic agents, such as Gadovist, Dotarem, and, occasionally, Multihance, were detected, while Omniscan was absent.^40^

Most published studies on Gd speciation preservation have employed frozen storage at – 20 to – 30 °C for wastewater, sewage, and surface water samples prior to speciation analysis.^10,17,37,39,41,47^ Our results, however, indicate that freezing offers no additional preservation benefit and may introduce freeze-concentration effects during sample handling. For example, a frozen vial of DI water in week 4 cracked during freezing and showed reduced Omniscan recovery (marked as a star in Figure 4). This was likely due to uneven distribution in the partially frozen sample, which only became fully detectable after complete thawing, consistent with the total Gd measured (Sample C in Figure 3). In contrast, a vial frozen under the same conditions but stored for 8 weeks retained both total GBCAs and total Gd (Sample D in Figure 3). This result shows that the drop in Omniscan for Sample C was due to a handling error rather than freezing conditions.

### Behavior of GBCAs in Complex Natural Waters (River Water) at Three Storage Temperatures

GBCA recovery in the river matrix was characterized by high stability of macrocyclic agents and dissociation of the linear chelate, with temperature-related effects most pronounced in river water and further influenced by filtration conditions (Figure 6).

**Figure 6:**
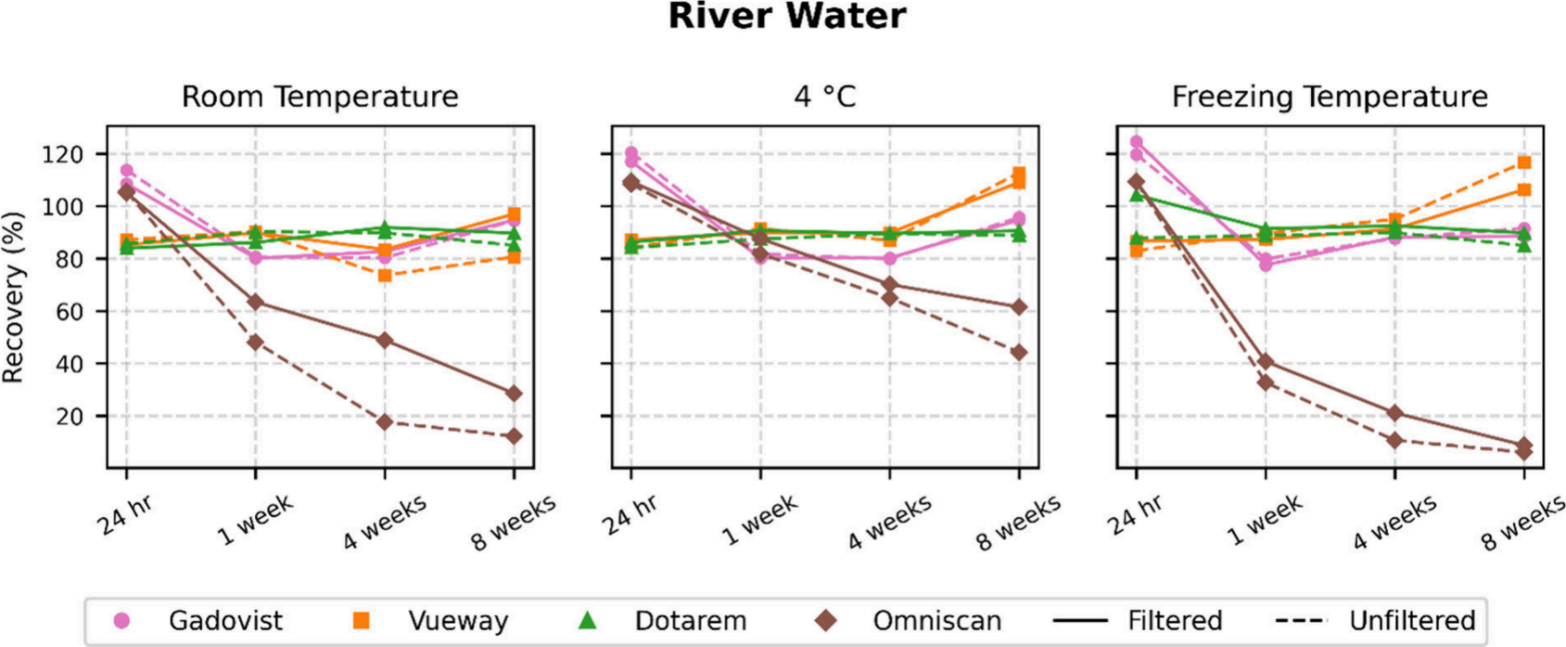
Stability of unacidified GBCAs in river water over time and storage temperatures. Filtration effects were most pronounced for the linear chelate, with higher recoveries observed in filtered samples.

### Effect of Temperature

In river water, Omniscan degraded rapidly, with recovery at room temperature reaching 50–60% after 1 week and 17–30% by week eight. Freezing accelerated degradation, with recovery dropping to 40% at 1 week and reaching zero by Week 8, which may be related to freeze-concentration effects. In contrast, refrigeration at 4 °C improved short-term stability, maintaining 90% recovery at 1 week and 45–60% by Week 8. Macrocyclic agents were stable in river water under all temperature conditions and maintained high recovery throughout the study period. Studies have found that the dominant anthropogenic species of Gd in natural waters are typically Gadovist, Dotarem, whereas linear agents such as Omniscan and Multihance are rarely detected or occur only at trace levels.^36,37,39,41^

This temperature dependence is also evident when examining total GBCAs and total Gd (mass balance) in river samples preserved under the same conditions but stored at different temperatures (samples F–H in Figure 3). Refrigeration at 4 °C preserves both total Gd and total GBCAs closest to the expected spike, whereas at room temperature, both decline over time. Under freezing, total Gd appears balanced, but total GBCAs are underestimated, indicating that freezing introduces freeze concentration effects rather than preserving the true speciation.

### Effect of Matrix Complexity

Filtration provided additional insight into the roles of particulates and microbial activity. Omniscan concentrations declined in both filtered and unfiltered river water, with the effect more pronounced in unfiltered samples. After 8 weeks, filtered samples showed about 60% recovery at 4 °C, whereas unfiltered samples showed about 40% recovery (Figure 6). Similarly, at room temperature, filtered samples showed 20–30% higher recovery than the unfiltered samples. For frozen samples, the effect of filtration was less pronounced, as Omniscan losses due to freezing were already substantial. Macrocyclic agents were less affected by filtration. Gadovist, Dotarem, and Vueway remained stable in both filtered and unfiltered water.

The presence of particulate matter may have promoted the sorption of Omniscan. In unfiltered samples, microbial activity could have further accelerated loss by producing competing organic ligands or changing the redox conditions in the water that favor chelate destabilization. This interpretation agrees with laboratory studies showing that Gd readily forms associations with humic substances and that organic-bound complexes can coexist with free Gd depending on pH and ionic strength.^35^ Moreover, wastewater studies have demonstrated the appearance of unidentified anthropogenic Gd species likely formed through interaction with organic or inorganic ligands.^47^ Such interactions could help explain the stronger degradation observed in unfiltered river water compared to filtered samples.

In some cases, concentrations showed a slight rebound over time, with values decreasing initially and then increasing at later time points. For Gadovist, the rebound remained below the initial concentration, which may reflect partial reassociation of Gd with the chelate. For Vueway, concentrations rose above the initial spike, indicating an additional factor influencing measured recovery, the precise cause of which remains unclear. Still, the overall pattern indicates that both agents maintain stability over time in river water, although minor variations in measured recovery may occur.

### Recommended Preservation Protocols and Implications for Environmental Monitoring

Preservation protocols must balance analytical accuracy with practical constraints of field sampling and laboratory workflows. The following recommendations prioritize conditions that maintain speciation integrity for both linear and macrocyclic chelates with particular attention to the most labile species.

### Laboratory Calibration Standards

GBCA calibration standards prepared in DI water remain stable at room temperature (20–25 °C) or under refrigeration (4 °C) for at least 8 weeks, with recoveries consistently above 90% for all species tested. Freezing provides no preservation advantage.

### Environmental Samples (Tap and River Water)

For the preservation of environmental samples, the most reliable approach is to measure immediately after sampling. If storage is unavoidable, refrigeration of filtered samples offers the least compromise, and freezing is not recommended. However, measured GBCA concentrations will still underestimate the initial levels after 1 week, and the bias becomes more pronounced for linear chelates during more extended storage periods. For river water, sample filtration generally enhances GBCA recovery.

Under no circumstances should samples intended for GBCA speciation analysis be acidified. A separate fraction should be collected and acidified exclusively for total Gd determination, regardless of sample type.

### Implications for Source Tracking and Risk Assessment

Currently, there are no official guidelines or monitoring requirements from agencies like the Environmental Protection Agency (EPA), World Health Organization (WHO), or European Agencies (EU) for Gd in the environment, even though it has been recognized as an emerging contaminant. Accurate speciation data are essential for evaluating both the sources and environmental risks of anthropogenic Gd. The selective degradation of linear chelates during improper storage can create misleading impressions of GBCA source contributions—for example, a sample originally containing equal amounts of Omniscan and Gadovist might, after acidified storage, appear to contain only Gadovist, falsely suggesting exclusive use of macrocyclic agents. Because linear and macrocyclic chelates differ substantially in thermodynamic stability and propensity to release free Gd^3+^ under environmental conditions, accurate speciation is critical for assessing actual risk. When this artifact is compounded across multiple samples or studies, it obscures true usage patterns and prevents the identification of facilities or practices that contribute disproportionately to linear chelate contamination.

Beyond source tracking, preservation artifacts directly compromise toxicological risk assessment. Linear chelates are more prone to dissociation, particularly in the presence of competing cations or under acidic conditions, increasing the potential for release of toxic free Gd^3+^. Because free Gd^3+^ interferes with calcium-dependent physiological processes and has been associated with severe human health outcomes such as tissue deposition, nephrogenic systemic fibrosis, and neurological effects, differentiation between intact chelated species and released Gd^3+^ is essential for evaluating potential human exposure risks.^16,22,48,49^ Linear chelates are less stable than macrocyclic agents and therefore present a higher potential for Gd release and uptake by aquatic organisms and plants, which supports the existence of exposure pathways through bioaccumulation and food webs that are underestimated when linear agents are selectively lost during sample preservation.^50–52^ Preservation protocols that selectively eliminate linear chelates from the analysis underestimate both the magnitude and toxicological character of anthropogenic Gd contamination.

The current understanding of GBCA usage patterns underscores the urgency of these preservation considerations.^53^ From 2011 to 2024, Medicare beneficiaries’ Part B fee-for-service in the United States underwent an estimated 41 million GBCAs examinations, primarily MRI (71%) and Magnetic Resonance Angiography (29%), totaling approximately 221 million mL for brain MRI alone. Overall, annual Gd contrast use increased steadily, with a mean year-overyear growth of 3.5% between 2014 and 2019, followed by a COVID-19-related decline of 15.6% in 2020. Usage rebounded in 2021, rising 10.1%, and then stabilized through 2024. Although comprehensive post-2016 data are not publicly available, the 2016 FDA report indicated that linear agents still accounted for approximately 18% of pediatric hospital GBCA use in the United States, with agents such as Magnevist and MultiHance reported in 15% and 9% of sampled facilities, respectively.^54^ While Omniscan has been discontinued and macrocyclic agents now dominate clinical practice, legacy linear chelate contamination persists in environmental systems, and continued low-level inputs remain possible. The protocols developed here enable monitoring programs to distinguish intact GBCA complexes from dissociated Gd, supporting more refined risk assessments than total anthropogenic Gd measurements alone can provide.

### Emergent Applications to Hydrologic Tracer Studies

Beyond contamination assessment, anthropogenic Gd derived from GBCAs has demonstrated utility as a tracer of wastewater influence, water transport, and recharge processes in aquatic systems. Kraemer et al. (2024) showed that GBCA-derived Gd exhibits conservative behavior across estuarine and coastal gradients in the North Sea, with anthropogenic Gd anomalies tracing riverine inputs, water-mass mixing, and large-scale circulation pathways; this work established Gd as a far-field tracer of persistent and mobile wastewater-derived substances.^55^ Guo et al. (2025) reported that positive Gd anomalies (expressed as Gd/Gd*) serve as a reliable tracer of reclaimed-water recharge in multisource artificial recharge systems, with greater robustness than conventional tracers such as Cl^–^ and artificial sweeteners and with quantification of recharge extent, flow paths, and groundwater mixing relationships.^56^

In addition to total anthropogenic Gd signals, GBCA speciation may serve as a tracer of water movement and recharge time scales. Different contrast agents were introduced to clinical practice at different times—for example, Vueway was approved in 2022, making its presence in groundwater indicative of recent (<5 year) recharge. Similarly, the presence or absence of linear agents, such as Omniscan (which has been phased out since 2013 in favor of macrocyclic alternatives), may provide temporal context for contamination events.

However, such tracer applications require careful consideration of differential environmental persistence. The absence of linear agents could reflect shifts in usage, preferential environmental degradation, or sample preservation artifacts. The stability data presented here inform the interpretation of such patterns and establish hold times suitable for reliable tracer studies.

Successful application of GBCA speciation as a temporal tracer will require: (1) validation in age-dated groundwater or sediment core samples with independent chronological constraints, (2) systematic documentation of regional usage patterns over time, (3) field-based degradation studies to establish environmental half-lives for different chelate structures, and (4) rigorous adherence to preservation protocols that maintain speciation integrity during sample transport and storage. These validation steps remain priorities for future research.

Environmental concentrations of anthropogenic Gd are generally low and highly variable compared to laboratory conditions, which makes it difficult to assess recovery under different sampling and preservation conditions using ambient samples alone. Controlled spike experiments at defined concentrations were therefore used to systematically evaluate changes and quantify recovery under the conditions studied. Starting from a known concentration allows detection of alterations that might remain hidden at environmental levels. In real-world waters, GBCA concentrations vary with the degree of contamination and may sometimes approach analytical detection limits. The sampling and preservation conditions tested remain the same regardless of environmental or Gd concentrations. Site-to-site differences in water chemistry, including variations in organic matter and major cations, can influence Gd behavior. Reporting key geochemical parameters is essential when applying these findings to environmental samples.

## Conclusion

The preservation protocols established here provide a foundation for standardized Gd speciation analysis in environmental monitoring. By minimizing storage-induced artifacts and maintaining the integrity of both linear and macrocyclic chelates, these practices will improve data quality, enable meaningful interstudy comparisons, and support more accurate assessments of anthropogenic Gd behavior in aquatic systems. Future work should validate these protocols across additional environmental matrices (e.g., groundwater, wastewater, sediment pore waters) and at lower concentrations typical of pristine waters distant from point sources. Adoption of these standardized practices across the research community will strengthen our collective understanding of GBCA environmental fate and inform evidence-based risk management strategies.

## Supplementary Material



## Notes

Additional experimental details, IC-ICP-MS and ICP-MS operation conditions, and literature summary of GBCA preservation methods across environmental matrices (PDF)
