# Supplementary material for: Preserving Gadolinium Speciation in Environmental Waters: Establishing Hold Times and Storage Protocols for Reliable Analysis
Source: ACS ES T Water. 2026 May 26;6(6):3842–51. doi: 10.1021/acsestwater.6c00241 (PMC13270507; doi:10.1021/acsestwater.6c00241)
Supplement: Supplementary file 1 [file ew6c00241_si_001.pdf]

Supporting Information

Preserving Gadolinium Speciation in Environmental Waters: Establishing Hold Times and Storage Protocols for Reliable Analysis

Ahmad Ezzaldine[1], Malsha Kanaththage[2], Gayan Rubasinghege[2], Bonnie Frey[3], Rachel Coyte[*1]

[1]New Mexico Institute of Mining and Technology, Department of Earth and Environmental Science, Socorro, NM, USA

[2]New Mexico Institute of Mining and Technology, Department of Chemistry, Socorro, NM, USA

[3]New Mexico Bureau of Geology and Mineral Resources, Socorro, NM, USA

***Correspondence:** Rachel Coyte

rachel.coyte@nmt.edu

Contents

S1. River Matrix Sampling Information 2

S2. GBCAs Information 2

S3. Chromatographic Injection and Flow Conditions 2

S4. Calibration and Data Processing 3

S5. GBCA Selection and Chromatographic Resolution 3

S6. Recovery Experiments and Stability Evaluation 3

S7. Data Availability 4

S8. Tables and Figures 5

References 12

S1. River Matrix Sampling Information

The river sample was collected on April 28, 2024, at noon. Measured field parameters included temperature 7.7 °C, pH 8.2, and specific conductance 428 µS. The river water was spiked with GBCAs shortly after sampling.

S2. GBCAs Information

Initially, 1000 µg/L stock solutions of GBCA were prepared using clinically available samples (Gadavist® 1mmol/mL, Dotarem® 0.5 mmol/mL, Omniscan® 0.5 mmol/mL, and Vueway® 0.5 mmol/mL) and diluted accordingly with purified water (18.2 MΩ, MilliporeSigma® Synergy® Ultrapure Water Purification System). Relevant information about the GBCAs used is presented in Table S2. Gd^{3+} stock solution prepared using high purity standard of 1000 mg/mL purchased from Inorganic Ventures.

S3. Instrument Setup

For each run, 12,000 µL of diluted solution was injected onto the column at 30,000 µL/min, while a consistent flow rate of 1,000 µL/min was maintained through the system. The mobile phase consisted of a 0.424 mM ammonium nitrate buffer, adjusted to pH 9.2 using sodium hydroxide and trace-metal grade nitric acid. A single-step gradient elution was applied, and the total chromatographic run time was 4 minutes, followed by a 100-second reset to wash the system and re-equilibrate the column. Each sample was analyzed as an instrumental triplicate. To reach maximum sensitivity, the ICP-MS was tuned prior to each analytical run using a 1 µg/L solution of lithium-7, yttrium-89, cerium-140, and thallium-205 prepared by diluting a multi-element tuning standard (Claritas PPT Tuning Solution 1, SPEX CertiPrep, Metuchen, NJ, USA; Cat# CL-TUNE-1; 10 mg/L stock in 2% HNO_3/5% HCl), manufactured under a UL ISO 9001 quality assurance system. The cerium oxide rate is maintained below 2.5%.

The standard setup of the Agilent 7900 ICP-MS uses a quartz torch, a quartz spray chamber at 2 °C, and a borosilicate glass Scott-type concentric nebulizer. Different nebulizers were employed depending on the analytical mode: a borosilicate glass concentric MicroMist nebulizer was used for total REE (total Gd) analysis, whereas a MicroFlow PFA-ST nebulizer was used during IC–ICP-MS coupling for GBCA speciation analysis. This configuration ensured optimal aerosol generation and signal stability for each analytical setup.

The ^{157}Gd and ^{158}Gd Gd isotopes are commonly used for sensitive and interference-free detection of Gd in complex matrices.

The instrumental limit of detection (LOD) for ^{157}Gd was 0.0031 µg/L in no-gas mode and 0.0022 µg/L in He collision mode. Relative standard deviations (RSDs) of integrated peak areas were consistently below 5% across replicate injections. During IC–ICP-MS operation, stable analyte signals were confirmed by maintaining RSDs at or below 3%, indicating

acceptable instrumental performance and signal stability. Overall sample-to-sample variability remained below 5% under optimized operating conditions.

Analytical and chromatographic characteristics (retention time,LOD,LOQ, MDL) of GBCAs are presented in Table S3.

S4. Data Processing

Calibration curves were prepared using a 50 µg/L stock solution of four GBCAs, serially diluted at factors ranging from 10× to 200×, yielding standard concentrations between 0.25 and 5 µg/L. Each calibration curve consisted of six points with R^2 values greater than 0.995. Sample preparation and injection were controlled using metal-free syringe pumps to prevent contamination. Data was processed using ESI Xceleri software (v1.3.1.59), and exported to a structured spreadsheet environment for quantitative analysis. The numerical data underlying the calibration curves, including final concentrations, are provided in the Supporting Information Excel file (Sheet2: "Calibration&Final Concentration") and are available for download at Zenodo (DOI: 10.5281/zenodo.17904805) as described in S7.

S5. GBCA Selection and Chromatographic Resolution

Gadovist, Dotarem, Omniscan, and Vueway were selected as this study's GBCAs to reflect a range of molecular structures and charges (macrocyclic vs. linear; ionic vs. nonionic), as these properties influence both their environmental persistence and kinetic stability. Each compound eluted at a distinct retention time, producing a unique peak in the Gd signal detected by ICP-MS (Figure S2). The area under each peak was integrated and compared to external calibration curves to quantify the concentration of each GBCA in the samples.

S6. Recovery Experiments and Stability Evaluation

Municipal tap and DI waters were not filtered, as they contain minimal suspended matter, and filtration was not expected to affect Gd speciation in these matrices significantly. The pH of acidified samples was verified using pH paper.

Since each of the DI, tap, and river samples contained known spiked concentrations (5 µg/L each) of the four GBCAs, we used this approach to assess how much of the original spiked concentration could be recovered after storage under different preservation conditions (the different preservation conditions applied to samples at each time point are presented in Figure S1 and Table S4). The spiked bulk matrices were mixed thoroughly to ensure homogeneity, then subsampled and stored in 50-mL polypropylene (PP) centrifuge tubes to generate individual experimental aliquots for analysis. Recovery analysis was conducted to evaluate the stability of each GBCA under the applied scenarios and to identify the factors that best preserve the original chemical composition of the samples. By comparing the measured concentrations of individual GBCAs to their known spiked levels, we estimated the extent of compound degradation, loss, or transformation across treatment scenarios. These results

inform the selection of appropriate preservation strategies for accurate trace-level gadolinium quantification in various sample matrices. Recovery percentage was calculated by comparing the measured GBCA concentration of each sample to the expected concentration, which was determined using the measured ICP-MS stock values together with the actual dilution ratios. This expected concentration is slightly different from the nominal 5 ppb target due to small variations in weighing, pipetting, and differences between the nominal and true stock concentrations (Table S2). To account for background Gd in the source water, total Gd was measured to prevent overestimation of GBCA-derived signals.

S7. Data Availability

The full raw IC-ICP-MS and ICP-MS dataset, calibration data and plots, and statistical tests have been deposited in Zenodo and can be accessed at

https://doi.org/10.5281/zenodo.17904804, https://doi.org/10.5281/zenodo.17904805

S8. Tables and Figures

Table S1. Summary of. preservation methods reported in previous literature on GBCAs speciation across different environmental matrices

Citation	Matrix	Analytes	Speciation Instrumentation	Conditions (bottle type, temp)	Speciation LOD/LOQ	Key findings and challenges
(Hennebrüder et al., 2004)	GBCA-spiked DI water	Gd^{3+} Magnevist	IC-ICP-TOF-MS	Polyethylene flasks were used to store river samples at 4 °C that did not undergo speciation. Storage conditions for the lab-prepared deionized water solution were not specified.	N/A	Gd–DTPA is stable at neutral pH but dissociates to Gd^{3+} at low pH, which improves recovery during analysis. Many sorbents retain free Gd^{3+} well but do not efficiently recover intact complexes, so there is a trade-off between measuring total Gd and preserving its original form.
(Krüger et al., 2004)	Synthetic purified Gd-tagged modular contrast agent in aqueous solution	Gd (free and complexed), intact DTPA-PNA-peptide complex	SEC-ICP-MS, NanoESI-MS	SEC at pH 4.75, Samples lyophilized (freeze-dried) and resolubilized before analysis.	N/A	A dual approach using nanoESI-MS and SEC-ICP-MS was applied to characterize the MRI agent for both functional and safety reasons. NanoESI-MS confirmed Gd presence through broadened isotope patterns but could not quantify saturation, while SEC-ICP-MS measured Gd saturation (~55–84%) and separated intact complexes from free Gd^{3+}. Some metal exchange occurred, with Fe^{3+} replacing Gd^{3+}, particularly after lyophilization.
(Loreti & Bettmer, 2004)	Human urine, hair, saliva, sweat after intravenous administration of Gd–DTPA	Gd^{3+} Magnevist	SEC–ICP–MS	Samples stored in polypropylene containers; urine, sweat, and saliva stored at –20 °C; hair at room temp; analysis performed at room temp; urine diluted in Tris-HCl (pH 7.4) and filtered (0.45 µm); 2-mercaptoethanol added to complex free Gd^{3+}	N/A	Gd–DTPA was the only form detected in urine, with about 70% excreted within 3 hours and over 99% within 24 hours, while no gadolinium appeared in saliva or sweat. Hair sampled four weeks later showed trace Gd, but harsh TMAH extraction partially broke down Gd–DTPA, and milder Tris-HCl methods failed to extract it. No interactions were seen with hemoglobin or transferrin, and the method allowed element- and isotope-specific speciation, though results came from a single patient..
(Kautenburger et al., 2006)	Diluted Gd^{3+} and Eu^{3+} solutions including DI water, iodinated humic acid, and background electrolytes were used to simulate environmental conditions.	Free Gd^{3+} Gd–humate complexes	CE – ICP-MS	10 mL tubes, mixed for 72 h at 25 °C in a rotator.	N/A	While Gd^{3+} was included in the experiments, CE–ICP–MS results and electropherograms were shown only for Eu. No data were presented for Gd speciation behavior.

(Kautenburger & Beck, 2007)	Eu- and Gd-spiked iodinated humic acid solutions (25 mg/L HA, 10 mM $NaClO_4$)	Free Gd^{3+}; Gd–humate complexes	UF–ICP–MS<br>CE–ICP–MS	The samples were mixed in a rotator for 72 h at 25 °C before CE injection, under different pH (4–9)	N/A	CE–ICP–MS separated and detected both free and humic-bound gadolinium in a single run, using iodine-tagged HA to track the complexes. Ultrafiltration caused some error as HA and its complexes partly passed through the filters.
(Künnemeyer et al., 2008)	Human blood plasma from 10 MRI patients, five treated with Magnevist and five with Gadovist.	Magnevist<br>Gadovist<br>Dotarem<br>Omniscan<br>Multihance	HILIC-ESI-MS, validated with ICP–OES	Plasma was collected in tubes with Liquemin, stored at room temperature <1 h, then centrifuged. Plasma stored at –30 °C; thawed to 21 °C; proteins precipitated with acetonitrile; centrifuged at 4 °C.	N/A	All five GBCAs were separated and detected in standard solutions, but only Magnevist and Gadovist were analyzed in plasma according to patient dosing. No degradation or transformation occurred, and only the administered GBCA was observed.
(Kahakachchi & Moore, 2009)	Diluted GBCA solutions including:<br>• DI Water<br>• Spiked human serum	Optimark<br>Magnevist<br>Prohance<br>Omniscan<br>Dotarem<br>Multihance	S-ICP-OES as primary detection. HPLC–ESI–MS as a secondary confirmation of chelate identity.	Volumetric glassware is used for all dilutions. DI water sample storage and temperature during analysis not specified. Serum stored at −20 °C, thawed at room temperature, diluted and 0.45 µm filtered.	LOD: 8 to 35 ng/ml<br>LOQ: 26 to 116 ng/ml	Over 99% of gadolinium in commercial formulations remained as intact complexes, though MultiHance showed up to four chromatographically separated isomers. HPLC–ESI-MS confirmed molecular ions for all GBCAs, and spiked human serum tests showed high recovery with minimal variability and no matrix interference.
(Kesava Raju et al., 2010)	Surface water, wastewater	Magnevist<br>Gadovist<br>Omniscan<br>Dotarem<br>Multihance	HILIC-ICP-MS	Polypropylene bottles, filtered (0.45 µm)	LOD: 22 ± 5 ng/L	The method separated five Gd–MRI complexes in environmental waters at trace levels. Magnevist, Dotarem, and Gadovist were the main species. Other linear agents were mostly absent. Total Gd exceeded the sum of chelates and indicated free or uncharacterized forms.
(Künnemeyer et al., 2009)	Hospital wastewater, municipal sewage, WWTP effluent	Gadovist<br>Multihance<br>Dotarem<br>Magnevist<br>Omniscan	HILIC-ICP-MS	Silylated glassware, filtered (0.45 µm), frozen at -30°C before analysis	LOD: 1×10^{-9} mol/L<br>LOQ: 6.6 × 10-10 mol/L	The method separated five GBCAs in 30 minutes per run. Silylated glassware prevented adsorption losses and filtration did not affect standards. Gadovist appeared in all samples while Dotarem and Multihance were lower in WWTP effluent because of differences in use and stability. GBCA levels decreased from the sewage system to the WWTP outlet but Gadovist remained detectable. Total Gd exceeded the sum of intact complexes and indicated the presence of free or transformed forms though no direct evidence of transformation was found.

(Telgmann et al., 2012)	Wastewater influent, effluent, activated sludge batch experiment with Gd–DTPA spike), chamber filter press effluent	Gadovist<br>Dotarem<br>Magnevist	HILIC–HPLC–ICP-SFMS<br>ICP-MS, IC–ICP-MS	PP bottles, 2h/24h composite samples, frozen at –30°C, filtered (0.2 µm) before analysis	LOD: 0.13 µg/L<br>LOQ: 0.43 µg/L	About 90% of Gd passed through the WWTP unchanged and 10% was removed by adsorption to activated sludge. Influent and effluent were dominated by Gd-BT-DO3A with smaller amounts of Gd-DTPA and Gd-DOTA. Chamber filter press effluent contained Gd-BT-DO3A, Gd-DOTA, and three unknown Gd species, indicating transformation during anaerobic sludge treatment. Some complexes in the effluent did not come from MRI agents and were absent in the influent. The method allowed fast speciation of five GBCAs at low detection limits, but the unknown species remain uncharacterized.
(Lindner et al., 2013)	Surface water,<br>Plants (river plants and experimental cress, Lepidium sativum)	Gadovist<br>Multihance<br>Dotarem<br>Magnevist<br>Omniscan	HILIC–ICP-MS	Water: collected in polypropylene bottles, filtered (0.20 µm), stored in the dark at 3 °C; preconcentrated by evaporation (1:20).<br>Plants: greenhouse-grown cress, exposed to GBCA solutions, extracted via ultrasonic sonication in water.	LOD = 51 ± 11 ng/L,<br>LOQ = 153 ± 34 ng/L (standards).<br>After preconcentration, LOD = ~2 ng/L,<br>LOQ = ~7 ng/L.	WWTP effluent caused a strong Gd anomaly in the receiving channel and total Gd increased from ~50 ng/L upstream to ~990 ng/L at the outlet. Speciation showed intact Gd-DOTA and Gd-BT-DO3A at ~456 and ~471 ng/L and concentrations dropped to ~65 ng/L downstream due to dilution. Chromatograms showed no new peaks and confirmed the complexes were stable. River plants contained Gd matching Gd-DOTA and extraction recovered 54 to 106 percent of the GBCAs. Experiments with cress showed uptake through roots and transport to leaves with little modification and no net accumulation.
(Birka et al., 2013)	Surface water	Gadovist<br>Multihance<br>Dotarem<br>Magnevist	HILIC–ICP-SFMS<br>with Apex Q desolvation	Collected in PE bottles, cooled <10 °C, frozen at −30 °C; filtered through 0.2 µm PTFE filters	LOD: 0.08-0.1 nmol/L<br>LOQ: 0.28-0.37 nmol/L.	Gadovist, Dotarem, and Magnevist were present in almost all samples and accounted for 74 to 89 percent of total Gd. The ratios between these agents remained stable across samples, showing that the complexes persist in the environment and resist degradation or transmetallation. Some Gd may exist in colloid-bound or unidentified forms. HILIC–ICP-SFMS with desolvation measured intact contrast agents at nanomolar levels without pre-concentration and improved detection limits while reducing analytical bias.
(Lindner et al., 2015)	Tap water	Gadovist<br>Multihance<br>Dotarem<br>Magnevist<br>Omniscan	ZIC-cHILIC-ICP-MS (with Pr-DOTA internal standard)	Samples in PP tubes, stored at 3 °C in dark, not acidified; filtered 0.2 µm; analyzed within 2 weeks	LOD: 1.4–3.5 ng/L;<br>LOQ: 4.7–11.7 ng/L	The ZIC-cHILIC column improved retention and sensitivity compared to earlier methods. The internal standard Pr-DOTA corrected for instrument drift and injection differences but did not address transmetallation of linear Gd complexes. Gadovist, Multihance, and Dotarem were detected in Berlin tap water at 10–20 ng/L, confirming their transport from WWTPs through rivers and bank filtration into drinking water.
(Birka et al., 2016)	Surface water and drinking water	Gadovist<br>Multihance<br>Dotarem<br>Magnevist	HILIC–ICP-MS with ultrasonic nebulization	Samples collected in PP vessels, cooled <10 °C immediately, filtered (0.2 µm PTFE)	LOD: 8–14 pmol/L<br>LOQ: 26–46 pmol/L	The method increased sensitivity tenfold over pneumatic nebulization. Magnevist, Dotarem, and Gadovist were detected in surface and drinking water, while Multihance was absent. Concentrations remained through all purification steps and no free Gd^{3+} or transformation products were found. Total Gd slightly exceeded the summed GBCAs due to low levels of Gd-BT-DO3A, not chemical transformation.
(Okabayashi et al., 2021)	Surface water	Gadovist<br>Dotarem<br>Magnevist<br>Omniscan<br>Eovist<br>Prohance	HILIC-ICP-MS	PP bottles, frozen storage, filtered (0.45 µm, 1 µm)	LOD: 3.4–22 ng/L;<br>LOQ: 11–72 ng/L	Aqueous HILIC-ICP-MS separated six GBCAs without organic solvents. Dotarem and Gadovist appeared near the WWTP effluent and all GBCAs were below detection 2000 m downstream. Two unknown Gd peaks were present. Free Gd^{3+} does not elute. Spiking Gd^{3+} increased the 150 s peak and produced Gd-DTPA, showing that released Gd^{3+} binds other ligands in the effluent. About 97 % of Gd at the WWTP site came from human sources. Identified GBCAs explained 71 % and the remainder came from transformed or unknown species. This shows that GBCA dissociation and transformation occur during wastewater treatment.

(Macke et al., 2021)	Surface water	Gadovist<br>Dotarem<br>Prohance<br>Magnevist<br>Multihance	Automated IC–ICP-MS	PE, cooled immediately (<10 °C), frozen at −20 °C until analysis, thawed at room temp, then filtered (0.2 µm PTFE)	LOD: 11–19 pmol/L<br>LOQs: 38–65 pmol/L.	A fast method separated five GBCAs in under two minutes without organic solvents and with minimal preparation. Macrocyclic GBCAs were present in most samples, while linear GBCAs were rarely detected or below LOQ. Later seasonal samples showed lower total Gd, probably from winter dilution. An unknown Gd species appeared in the Ems River at 57 s and made up to 13.9 % of total Gd. Its structure is unknown but may be an anionic complex from degradation or metal exchange.
(Athmer et al., 2025)	Surface water	Gadovist<br>Dotarem<br>Prohance<br>Magnevist<br>Multihance<br>Vueway	Automated IC–ICP-MS	Samples taken in PE bottles, cooled, frozen at −20 °C, filtered (0.45 µm)	LOD: 0.8 –5.8 pM<br>LOQs: 2.7–19.5 pM	The method detected Vueway with high sensitivity and showed that macrocyclic GBCAs are stable while linear ones recover poorly at low levels. Gadovist, Prohance, and Dotarem dominated anthropogenic Gd, with other GBCAs at trace levels or absent. An unknown Gd species appeared occasionally, possibly from GBCA breakdown, treatment processes, or free Gd^{3+} re-complexation.

Table S2. Spike–Dilution Factors and Expected Concentrations for GBCA Recovery Experiments in Different Water Matrices.

GBCA Name	Sample Matrix	Spike Volume Added (mL)	Total Sample Volume After Spike (mL)	Measured Stock Concentration (µg/L)	Expected Concentration in Sample (µg/L)
Gadovist	DI	15.1	2998.3	988.7	5.0
Gadovist	River	15.1	3012.1	988.7	4.9
Gadovist	Tap	15.1	2977	988.7	5.0
Dotarem	DI	14.9	2991.1	922.2	4.6
Dotarem	River	15.0	2982.9	922.2	4.6
Dotarem	Tap	14.9	3011.4	922.2	4.5
Omniscan	DI	15.0	2987.3	1019.5	5.1
Omniscan	River	15.7	3014.3	1019.5	5.3
Omniscan	Tap	15.0	2996.4	1019.5	5.1
Vueway	DI	14.7	2989.4	981.1	4.8
Vueway	River	14.8	3007.3	981.1	4.8
Vueway	Tap	15.0	2992.5	981.1	4.9

Table S3. Analytical and Chromatographic Characteristics of GBCAs

GBCA Brand Name	GBCA Generic Name	Manufacturer	Retention Time (sec)	LOD (µg/L)	LOQ (µg/L)	MDL (µg/L)	Structure
Omniscan	Gadodiamide	GE HealthCare	24 +/- 10	0.0028	0.0085	0.0570	Linear, non-ionic
Gadovist	Gadobutrol	Bayer	47 +/- 8	0.0034	0.0103	0.0501	Macrocyclic, non-ionic
Vueway	Gadopiclenol	Bracco Diagnostics	63 +/- 8	0.0103	0.0311	0.0237	Macrocyclic, non-ionic
Dotarem	Gadoterate meglumine	Guerbet	115+/- 10	0.0035	0.0105	0.0265	Macrocyclic, ionic

Table S4. The table shows the experimental design for a single representative time point, resulting in 24 sampless2. Each preservation condition was evaluated at four time points (24 hours, 1 week, 4 weeks, and 8 weeks), resulting in a total of 96 samples (24 conditions × 4 time points).

Matrix	Filtration	Acidification	Temperature	Sample condition description
Deionized water	Unfiltered	Unacidified	Room temperature	Deionized water stored at room temperature
			4 °C	Deionized water stored at 4 °C
			−20 °C	Deionized water stored frozen at −20 °C
		Acidified (pH < 2)	Room temperature	Deionized water acidified and stored at room temperature
			4 °C	Deionized water acidified and stored at 4 °C
			−20 °C	Deionized water acidified and stored frozen at −20 °C
Tap water	Unfiltered	Unacidified	Room temperature	Tap water stored at room temperature
			4 °C	Tap water stored at 4 °C
			−20 °C	Tap water stored frozen at −20 °C
		Acidified (pH < 2)	Room temperature	Tap water acidified and stored at room temperature
			4 °C	Tap water acidified and stored at 4 °C
			−20 °C	Tap water acidified and stored frozen at −20 °C
River water	Filtered	Unacidified	Room temperature	Filtered river water stored at room temperature
			4 °C	Filtered river water stored at 4 °C
			−20 °C	Filtered river water stored frozen at −20 °C
		Acidified (pH < 2)	Room temperature	Filtered river water acidified and stored at room temperature
			4 °C	Filtered river water acidified and stored at 4 °C
			−20 °C	Filtered river water acidified and stored frozen at −20 °C
	Unfiltered	Unacidified	Room temperature	Unfiltered river water stored at room temperature
			4 °C	Unfiltered river water stored at 4 °C
			−20 °C	Unfiltered river water stored frozen at −20 °C
		Acidified (pH < 2)	Room temperature	Unfiltered river water acidified and stored at room temperature

			4 °C	Unfiltered river water acidified and stored at 4 °C
			−20 °C	Unfiltered river water acidified and stored frozen at −20 °C

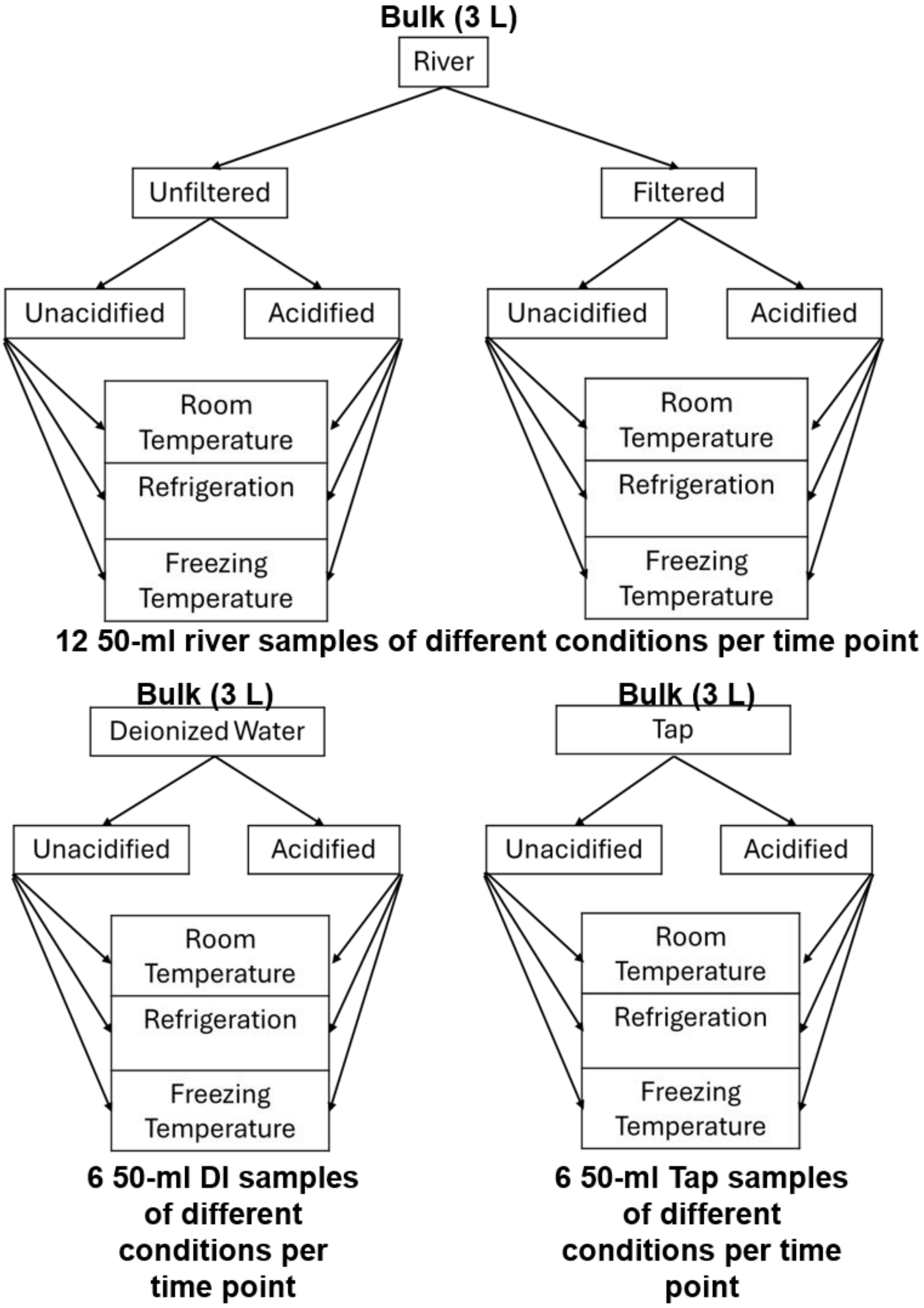

Figure S1. Flow Chart of the different preservation conditions applied to samples at each time point.

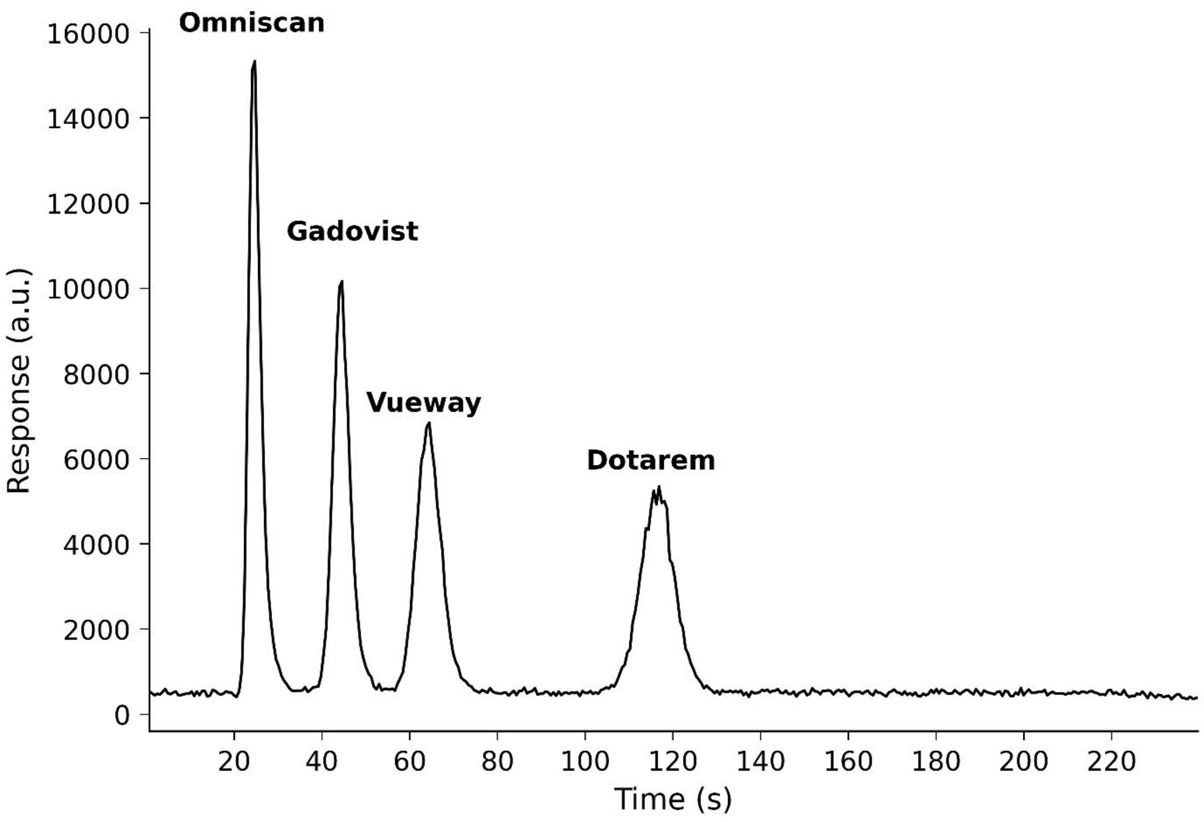

Figure S2. IC-ICP-MS Chromatogram Showing Distinct Retention Times of Selected GBCAs.

References

Athmer, M., Marotz, L., & Karst, U. (2025). Rapid and sensitive speciation analysis of established and emerging gadolinium-based contrast agents in the aquatic environment by IC-ICP-MS. *Journal of Analytical Atomic Spectrometry*, *40*(8), 2138–2149. https://doi.org/10.1039/d5ja00159e

Birka, M., Wehe, C. A., Hachmöller, O., Sperling, M., & Karst, U. (2016). Tracing gadolinium-based contrast agents from surface water to drinking water by means of speciation analysis. *Journal of Chromatography A*, *1440*, 105–111. https://doi.org/10.1016/j.chroma.2016.02.050

Birka, M., Wehe, C. A., Telgmann, L., Sperling, M., & Karst, U. (2013). Sensitive quantification of gadolinium-based magnetic resonance imaging contrast agents in surface waters using hydrophilic interaction liquid chromatography and inductively coupled plasma sector field mass spectrometry. *Journal of Chromatography A*, *1308*, 125–131. https://doi.org/10.1016/j.chroma.2013.08.017

Hennebrüder, K., Wennrich, R., Mattusch, J., Stärk, H. J., & Engewald, W. (2004). Determination of gadolinium in river water by SPE preconcentration and ICP-MS. *Talanta*, *63*(2), 309–316. https://doi.org/10.1016/j.talanta.2003.10.053

Kahakachchi, C. L., & Moore, D. A. (2009). Speciation of gadolinium in gadolinium-based magnetic resonance imaging agents by high performance liquid chromatography inductively coupled plasma optical emission spectrometry. *Journal of Analytical Atomic Spectrometry*, *24*(10), 1389–1396. https://doi.org/10.1039/b907044c

Kautenburger, R., & Beck, H. P. (2007). Complexation studies with lanthanides and humic acid analyzed by ultrafiltration and capillary electrophoresis-inductively coupled plasma mass spectrometry. *Journal of Chromatography A*, *1159*(1–2), 75–80. https://doi.org/10.1016/j.chroma.2007.03.092

Kautenburger, R., Nowotka, K., & Beck, H. P. (2006). Online analysis of europium and gadolinium species complexed or uncomplexed with humic acid by capillary electrophoresis-inductively coupled plasma mass spectrometry. *Analytical and Bioanalytical Chemistry*, *384*(6), 1416–1422. https://doi.org/10.1007/s00216-006-0299-3

Kesava Raju, C. S., Cossmer, A., Scharf, H., Panne, U., & Lück, D. (2010). Speciation of gadolinium based MRI contrast agents in environmental water samples using hydrophilic interaction chromatography hyphenated with inductively coupled plasma mass spectrometry. *Journal of Analytical Atomic Spectrometry*, *25*(1), 55–61. https://doi.org/10.1039/b919959d

Krüger, R., Braun, K., Pipkorn, R., & Lehmann, W. D. (2004). Characterization of a gadolinium-tagged modular contrast agent by element and molecular mass spectrometry. *Journal of Analytical Atomic Spectrometry*, *19*(7), 852–857. https://doi.org/10.1039/b315649d

Künnemeyer, J., Terborg, L., Meermann, B., Moller, I., Scheffer, A., & Karst, U. (2009). Speciation analysis of gadolinium chelates in hospital effluents and wastewater treatment plant sewage by a novel HILIC/ICP-MS method. *Environmental Science and Technology*, *43*(14), 5547–5548. https://doi.org/10.1021/es901406g

Künnemeyer, J., Terborg, L., Nowak, S., Scheffer, A., Telgmann, L., Tokmak, F., Günsel, A., Wiesmüller, G., Reichelt, S., & Karst, U. (2008). Speciation analysis of gadolinium-based MRI contrast agents in blood plasma by hydrophilic interaction chromatography/electrospray mass spectrometry. *Analytical Chemistry*, *80*(21), 8163–8170. https://doi.org/10.1021/ac801264j

Lindner, U., Lingott, J., Richter, S., Jakubowski, N., & Panne, U. (2013). Speciation of gadolinium in surface water samples and plants by hydrophilic interaction chromatography hyphenated with inductively coupled plasma mass spectrometry. *Analytical and Bioanalytical Chemistry*, *405*(6), 1865–1873. https://doi.org/10.1007/s00216-012-6643-x

Lindner, U., Lingott, J., Richter, S., Jiang, W., Jakubowski, N., & Panne, U. (2015). Analysis of Gadolinium-based contrast agents in tap water with a new hydrophilic interaction chromatography (ZIC-cHILIC) hyphenated with inductively coupled plasma mass spectrometry. *Analytical and Bioanalytical Chemistry*, *407*(9), 2415–2422. https://doi.org/10.1007/s00216-014-8368-5

Loreti, V., & Bettmer, J. (2004). Determination of the MRI contrast agent Gd-DTPA by SEC-ICP-MS. *Analytical and Bioanalytical Chemistry*, *379*(7–8), 1050–1054. https://doi.org/10.1007/s00216-004-2700-4

Macke, M., Quarles, C. D., Sperling, M., & Karst, U. (2021). Fast and automated monitoring of gadolinium-based contrast agents in surface waters. *Water Research*, *207*(October). https://doi.org/10.1016/j.watres.2021.117836

Okabayashi, S., Kawane, L., Mrabawani, N. Y., Iwai, T., Narukawa, T., Tsuboi, M., & Chiba,

K. (2021). Speciation analysis of Gadolinium-based contrast agents using aqueous eluent-hydrophilic interaction liquid chromatography hyphenated with inductively coupled plasma-mass spectrometry. *Talanta*, *222*(April 2020), 121531. https://doi.org/10.1016/j.talanta.2020.121531

Telgmann, L., Wehe, C. A., Birka, M., Künnemeyer, J., Nowak, S., Sperling, M., & Karst, U. (2012). Speciation and isotope dilution analysis of gadolinium-based contrast agents in wastewater. *Environmental Science and Technology*, *46*(21), 11929–11936. https://doi.org/10.1021/es301981z
